# Treatment of Sciatica Following Uterine Cancer with Acupuncture: A Case Report

**DOI:** 10.3390/medicines5010006

**Published:** 2018-01-15

**Authors:** Henry Xiao, Christopher Zaslawski, Janette Vardy, Byeongsang Oh

**Affiliations:** 1 School of Life Sciences, University of Technology Sydney, Ultimo, NSW 2007, Australia; chris.Zaslawski@uts.edu.au (C.Z.); byeong.oh@sydney.edu.au (B.O.); 2 Sydney Medical School, University of Sydney, Camperdown, NSW 2006, Australia; janette.vardy@sydney.edu.au; 3 Concord Cancer Centre, Concord Repatriation General Hospital, Concord, NSW 2137, Australia; 4 Northern Sydney Cancer Centre, Royal North Shore Hospital, St Leonards, NSW 2065, Australia

**Keywords:** uterine cancer, sciatica, acupuncture

## Abstract

For women, gynaecological or obstetrical disorders are second to disc prolapse as the most common cause of sciatica. As not many effective conventional treatments can be found for sciatica following uterine cancer, patients may seek assistance from complementary and alternative medicine. Here, we present a case of a woman with severe and chronic sciatica secondary to uterine cancer who experienced temporary relief from acupuncture.

## 1. Introduction

Uterine cancer (UC), also known as endometrial cancer, arises from the epithelium lining of the uterus [1]. The most common symptoms in women are abnormal uterine bleeding, vaginal discharge and nonspecific gastrointestinal symptoms [1]. On the other hand, the observed symptoms of the sciatica could be due to a number of factors, which we are unfortunately unable to confirm. However from a neuro-anatomical perspective, sciatica is generally caused by a disc herniation or spinal epidural abscess along the sciatic nerve, which anatomically contains fibres originating in the L4-S2 roots [1]. It is not uncommon for sciatica to result from the uterine tumour compressing the lumbosacral plexus, in particular the L4-L5 nerve roots [2]. The first reported case of this was in 1992 when a calcified degenerated myoma, suspected to be an ovarian tumour, induced sciatica by compressing the sciatic nerve in the lumbosacral plexus [3]. Uterine fibroids are another diagnostic consideration [4], and after pregnancy endometriosis is the most common cause of sciatica [2].

Acupuncture has been utilised to treat sciatic symptoms in China and in Western countries. Clinical trials have documented the effects of acupuncture on multiple cancer-related symptoms including pain, but the efficacy of acupuncture for managing cancer-related sciatic symptoms remains unclear [5]. Here, we report a case of acupuncture treating sciatica following uterine cancer.

## 2. Case Description 

The patient was a 66-year-old female diagnosed with a stage III, type 2 endometrial cancer in late 2013. Her past medical history included type 2 diabetes diagnosed in 2012 (treated with oral hypoglycaemics), asthma (managed with inhalers), and arthritis in her foot (treated with Glucosamine hydrochloride/Chondroitin sulphate 500 mg/400 mg and Paracetamol 665 mg as required). In addition she described her difficulties with concentrating and memory for which she took ginkgo biloba 2000 mg (1–2 tablets a day). 

The tumour was a serous, poorly differentiated, uterine adenocarcinoma that invaded more than 50% of the myometrial thickness, and involved the uterus and cervix. She was treated with a total abdominal hysterectomy and bilateral salpingo-oophorectomy. Post-operatively, she had 5 cycles of chemotherapy (Paclitaxel and Carboplatin), which was complicated by neutropenia and peripheral neuropathy. This was followed by 25 fractions of pelvic radiotherapy.

After surgery, although there were no recurrent symptoms of UC, the patient developed a dull pain down the left leg, radiating from the L5-S1 segments to the second toe. Standing and sitting exacerbated the pain. Before the tumour diagnosis, there were no sciatic symptoms. The sciatica therefore considerably impacted her quality of life.

In 2016, she was referred to the acupuncture clinic for individualised acupuncture treatments of her back and leg pain. The pain was described as a dull and radiating from the L5-S1 segments to her second toe, and had been present for three years. The patient attended 6 weekly sessions to have the acupuncture administered by a final year intern (HX) at a university-affiliated cancer centre that was supervised by a professional acupuncturist (BO). At each visit, the patient rated her pain from 0 to 10, with 0 being no pain and 10 being the worst conceivable pain. The patient initially reported a 3/10 pain at rest, worsening to 8/10 when she stood up or sat down.

**Figure 1:**
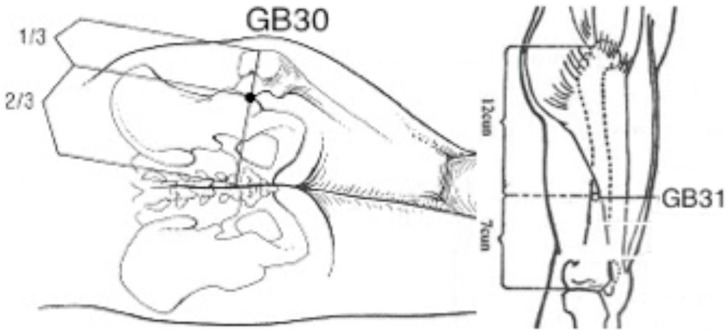
Acupuncture points GB30 and GB31.

The style of acupuncture conducted was Traditional Chinese Medicine. During the acupuncture treatment, a combination of local and distal points were needled to address the sciatica, as shown in Figure 1. Apart than the acupuncture, there were no other interventions. The Traditional Chinese Medicine (TCM) diagnosis was Qi Stagnation in the Gall Bladder Channel. 

With the patient lying on one side, the area was sterilised with an alcohol swab, the needles (30 and 0.12 mm gauge, Tempo J-Type Acupuncture Needles) were inserted at 90° until the patient experienced De Qi (a dull pressing and radiating sensation). The insertion for GB30 and GB31 for sciatica was 4–6 cm and 2–3 cm respectively [6] in the left hand side. The acupuncture points administered for her other signs and symptoms (e.g., shoulder pain, arthritis in her feet, poor concentration) were GB21, LI14, BL60, SP6, KD3 (first treatment), BL60, BL28, KD7, GB34, GB21 (second treatment), GB21, LU3, *Yintang*, LR3, *Erjian*, TE5 (third treatment), LI4, LI10, GB34, ST41, BL13, BL15, BL23 (fourth treatment), SP3, BL65, GB34, GV24, *Anmian* (fifth treatment), and GV20, LU7, GB34, LR3, KD6 (sixth treatment). The patient was left to rest for 30 min before the needles were removed. 

After one treatment, the patient reported a decrease in pain intensity, rating her pain as 1/10 at rest and 5/10 when she stood up or sat down. After the second treatment, she reported further improvement in pain and she was able to perform most of her daily activities, including sitting and standing. She was able to perform stretching exercises such as Qigong without experiencing severe sciatic pain. The patient expressed improved mood and an interest in continuing acupuncture to address her other pain symptoms such as her arthritis in her feet, so that she would be able to participate in low impact exercises. Treatments in weeks 3–5 focused on her right shoulder pain and arthritis in her feet. The patient experienced little to no sciatica during this time. During the follow-up assessment at week 12, she presented with sciatica again, and rated the pain to be 2/10 at rest, worsening to 5/10 when she stood up or sat down.

## 3. Discussion

Sciatica is usually treated with non-steroidal anti-inflammatory drugs, but there is a lack of consensus regarding the efficacy of these drugs [7]. As a result, some patients turn to acupuncture for which there is some evidence of efficacy in treating painful symptoms associated with sciatica [8]. Acupuncture has been shown to be minimally invasive [9] and safe [10]. The treatment modality of acupuncture is based on the principles of TCM. The classic text, ‘Song of Points for Miscellaneous Diseases’ from ‘The Glorious Anthology of Acupuncture and Moxibustion’ written during the Ming Dynasty, in China, indicates that the acupuncture points of GB30, GB31 and LR2 were used for lumbar pain radiating down the leg [11]. 

In this report, we document the earliest case, to our knowledge, of potentially decreasing the intensity of sciatica following uterine cancer with acupuncture. After two treatment sessions, our patient provided feedback that her pain had improved. She reported that not only did she experience less sciatic pain, but the acupuncture also greatly improved her mood, contributing to better quality of life with family and friends. Furthermore, her newly managed pain opened her up to consider acupuncture for addressing other symptoms such as foot arthritis, which had been a secondary complaint. Nevertheless, she did not have complete relief from her sciatic pain at the sixth treatment, suggesting that chronic conditions may require a longer-term pain-management plan.

There are limitations to our case report. Firstly, there is a possibility of the synergistic effect of acupuncture with her pharmaceutical intervention; however, this had been stable and long term. The pharmaceutical intervention in this case refers to the patient’s ongoing medications for type 2 diabetes, asthma, and foot arthritis. Furthermore, it may be that acupuncture plus lifestyle change could have improved the treatment efficacy. The very nature of a case report is that the clinical experience of only a single patient is presented; however, a testable hypothesis can be generated for future studies to investigate. Furthermore, the proximity between the tumour and sciatica events in the timeline leads to the conclusion that there could be a connection between the two. There exists the possibility that the patient’s sciatic pain originated partially from a problem that was musculoskeletal in nature. To confirm the effect of acupuncture on sciatica following uterine cancer would require a much larger study, controlling for confounding factors and bias, including the placebo effect.

## 4. Conclusions

Sciatica following uterine cancer can be a debilitating condition with few successful treatments. Our case report documents how a woman with severe and persistent sciatica pain that could have been a result of her uterine cancer experienced considerable relief from acupuncture. This warrants further evaluation to determine the efficacy of acupuncture for sciatica following uterine cancer.
